# Impact of prior anthracycline or taxane use on eribulin effectiveness as first-line treatment for metastatic breast cancer: results from two phase 2, multicenter, single-arm studies

**DOI:** 10.1186/s40064-015-1322-y

**Published:** 2015-09-21

**Authors:** Joyce O’Shaughnessy, Kristi McIntyre, Lee Schwartzberg, Sharon Wilks, Shannon Puhalla, Erhan Berrak, James Song, Linda Vahdat

**Affiliations:** Texas Oncology-Baylor Charles A. Sammons Cancer Center, US Oncology, 3410 Worth Street, Ste 400, Dallas, TX 75246 USA; Texas Oncology-Dallas Presbyterian Hospital, US Oncology, Dallas, TX 75231 USA; West Clinic, Memphis, TN 38120 USA; US Oncology-Cancer Care Centers of South Texas, San Antonio, TX 78217 USA; University of Pittsburgh Medical Center, Pittsburgh, PA 15213 USA; Eisai Inc., Woodcliff Lake, NJ 07677 USA; Weill Cornell Medical College, New York, NY 10065 USA

**Keywords:** Eribulin, Metastatic breast cancer, Prior chemotherapy, Objective response rate, Progression-free survival, Tolerability

## Abstract

Eribulin mesylate has efficacy in patients who have received ≥2 prior chemotherapies for metastatic breast cancer (MBC) including an anthracycline and taxane. Phase 2 trials showed clinical activity and acceptable tolerability of first-line eribulin (HER2− MBC; Study 206) and eribulin plus trastuzumab (HER2+ MBC; Study 208). Prespecified analyses evaluated efficacy by prior anthracycline and/or taxane use. Patients received eribulin mesylate (1.4 mg/m^2^ IV; Days 1 and 8) and, in Study 208, trastuzumab (8 mg/kg IV/Cycle 1, then 6 mg/kg; Day 1) in 21-day cycles. Endpoints included objective response rate (ORR), progression-free survival (PFS), and tolerability. In Study 206 (N = 56), 48 % of patients had received prior anthracycline, 46 % prior taxane, 36 % prior anthracycline and taxane, and 41 % were chemotherapy-naïve. In Study 208 (N = 52), these percentages were 21, 44, 17, and 52 %, respectively. In Study 206, ORR and median PFS were similar for anthracycline-pretreated (25.9 %, 5.8 months), taxane-pretreated (26.9 %, 5.8 months), anthracycline- and taxane-pretreated (25.0 %, 6.7 months), and anthracycline/taxane-naïve patients (30.4 %, 7.6 months). In Study 208, ORR/median PFS were 63.6 %/6.7 months among anthracycline-pretreated patients, 56.5 %/6.8 months among taxane-pretreated patients, 55.6 %/5.9 months among anthracycline- and taxane-pretreated patients, and 81.5 %/13.1 months among anthracycline/taxane-naïve patients. Tolerability was generally similar among subgroups. In these studies, first-line eribulin in HER2− MBC and eribulin/trastuzumab in HER2+ MBC was effective with acceptable tolerability, regardless of prior anthracycline/taxane treatment. Prior chemotherapy was associated with lower ORR and shorter PFS with eribulin/trastuzumab in HER2+ MBC but not with eribulin in HER2− MBC.

**Electronic supplementary material:**

The online version of this article (doi:10.1186/s40064-015-1322-y) contains supplementary material, which is available to authorized users.

## Background

In the US, approximately 5–10 % of women diagnosed with breast cancer (BC) have de novo metastatic disease and have an estimated 5-year survival rate of 24.3 % (National Cancer Institute 2014; American Cancer Society 2014). In addition, approximately 30 % of patients with stage I to III BC at diagnosis will subsequently develop metastatic disease (O’Shaughnessy 2005). Treatment selection for metastatic breast cancer (MBC) is guided by several factors, including patients’ choices, hormone receptor (estrogen receptor and progesterone receptor) expression, human epidermal growth factor receptor 2 (HER2) expression, site and burden of metastases, disease-related symptoms, prior treatment history, toxicity, comorbid conditions, patient age, and menopausal status, amongst others (National Comprehensive Cancer Network 2015; Partridge et al. 2014).

The overall rate of BC that overexpresses the transmembrane tyrosine kinase receptor, HER2, is 22.2 % as was demonstrated in a recent survey of 107 published studies involving 39,730 patients (Ross et al. 2009). Preferred single agents for MBC chemotherapy according to NCCN guidelines include anthracyclines, taxanes, antimetabolites (e.g., capecitabine and gemcitabine), and other microtubule disrupting agents (e.g., vinorelbine and eribulin) (National Comprehensive Cancer Network 2015). Current guidelines recommend sequential single-agent chemotherapy (in most cases) for HER2-negative (HER2−) MBC that is hormone receptor-negative or endocrine-resistant; however, there is not a single specific preferred chemotherapy agent or regimen (National Comprehensive Cancer Network 2015; Partridge et al. 2014). For women with HER2-positive (HER2+) metastatic disease, trastuzumab, a humanized monoclonal antibody directed against the extracellular domain of HER2 (Valabrega et al. 2007), combined with recently approved pertuzumab and a taxane (docetaxel or paclitaxel) are recommended as first-line therapy (National Comprehensive Cancer Network 2015; Giordano et al. 2014).

Eribulin is a non-taxane microtubule inhibitor that is a structurally modified synthetic analog of halichondrin B (Jordan et al. 2005; Okouneva et al. 2008; Smith et al. 2010; Jordan and Kamath 2007). Eribulin has a unique mode of cytotoxic action that is distinct from those of other tubulin-targeting agents; it binds and inhibits only the growing + ends, inhibiting the microtubule growth phase without affecting the shortening phase and causes tubulin sequestration into non-productive aggregates. This novel tubulin-directed mechanism may explain eribulin’s observed non-cross-resistant antitumor activity following taxane therapy (Jordan et al. 2005; Okouneva et al. 2008; Smith et al. 2010; Jordan and Kamath 2007; Jain and Cigler 2012).

In women with MBC who had previously received at least 2 chemotherapeutic regimens (including an anthracycline and a taxane) for metastatic disease, eribulin has demonstrated antitumor activity and improved overall survival (Vahdat et al. 2009; Cortes et al. 2010; Aogi et al. 2012; Cortes et al. 2011; Halaven^®^^2014^). Approval by the United States (US) Food and Drug Administration for eribulin in this setting was based on the results from the phase 3 EMBRACE study, where single-agent eribulin significantly improved overall survival (OS) in patients with MBC compared with treatment of physician’s choice (Cortes et al. 2011). A more recent phase 3 study, which compared eribulin and capecitabine as first-, second-, or third-line therapy for MBC in women who had previously received an anthracycline and a taxane, found no significant difference in OS between the 2 treatments [hazard ratio 0.88 (95 % CI 0.77, 1.00); p = 0.056]; however, for eribulin the median OS (15.9 months), median PFS (4.1 months), and objective response rate (11 %) were comparable to those achieved with eribulin in the EMBRACE trial (13.1, 3.7 months, and 12 %, respectively) (Cortes et al. 2011; Kaufman et al. 2015).

Due to its antitumor activity in the challenging setting of late-line treatment, infusion schedule (1.4 mg/m^2^ IV over 2–5 min on Days 1 and 8 of a 21-day cycle), and lack of premedication requirement to prevent hypersensitivity, assessment of eribulin in the first-line setting for women with MBC was of clinical interest (Cortes et al. 2011; Halaven^®^^2014^). Results from two, single-arm, multicenter, phase 2 trials, Study 206 (eribulin monotherapy in HER2− MBC) and Study 208 (combination eribulin plus trastuzumab in HER2+ MBC), as first-line treatment in patients with locally recurrent BC or MBC have been published (McIntyre et al. 2014; Wilks et al. 2014). In Study 206 (N = 56), the objective response rate (ORR) with single-agent eribulin was 29 %, median duration of response (DOR) was 5.8 months, and median progression-free survival (PFS) was 6.8 months (McIntyre et al. 2014). In Study 208 (N = 52), ORR with eribulin/trastuzumab was 71.2 %, median DOR was 11.1 months, and median PFS was 11.6 months (Wilks et al. 2014). Because many patients receive an anthracycline as adjuvant or neoadjuvant (neo/adjuvant therapy), we conducted a prespecified analysis of both trials to examine efficacy of these regimens based on prior anthracycline and/or taxane pretreatment.

## Methods

### Study design

Complete trial methodology for the 206 and 208 studies have been previously published (McIntyre et al. 2014; Wilks et al. 2014). Both were multicenter, phase 2, open-label trials that assessed eribulin as first-line treatment in patients with HER2− locally recurrent BC or MBC not previously treated with chemotherapy in the metastatic setting (Study 206) or eribulin combined with trastuzumab treatment in patients with HER2+ locally recurrent BC or MBC (Study 208). Both studies were conducted in accordance with the Declaration of Helsinki, and the protocols and informed consent forms were approved by institutional review boards. All patients for both studies provided written informed consent before undergoing any study-related procedures.

### Endpoints

Prespecified analyses included evaluation of the primary and secondary endpoints of ORR and PFS, respectively, according to prior neo/adjuvant anthracycline or taxane pretreatment. For both trials, the primary efficacy variable was ORR, defined as the proportion of patients who achieved an overall best response of complete response (CR) or a partial response (PR). PFS was measured from the start of treatment until disease progression or death from any cause. The censoring rules for PFS were according to FDA guidance in 2007. Tumor assessments were performed every 6 weeks in the first 6 cycles and every 12 weeks thereafter using RECIST v1.1.

### Safety/tolerability

Safety evaluations at baseline and subsequent visits included adverse events (AEs), clinical laboratory tests, physical examination, vital signs, and electrocardiogram assessments. Treatment-emergent AEs (TEAEs) were defined as AEs that started or worsened after the first dose of study treatment.

### Statistical analysis

The statistical methods have been published with the primary analyses for Studies 206 and 208 (McIntyre et al. 2014; Wilks et al. 2014). Efficacy analyses were based primarily on the full analysis set (FAS), which included all patients who received ≥1 dose(s) of study treatment. The safety analysis set included all patients who received ≥1 dose(s) of eribulin and had ≥1 postbaseline safety evaluation. All efficacy endpoints were summarized descriptively. Kaplan–Meier method was used to estimate the time to event variables (e.g., PFS). Greenwood method was used to construct 95 % confidence interval (95 % CI) for the median. Exact method was used to construct 95 % CI for rate variables (e.g., ORR). Statistical analyses and summaries were performed using SAS for Windows v. 9.3.

## Results

### Baseline characteristics and demographics

In Study 206 (single-agent eribulin in HER2− MBC), 56 female patients were enrolled, of which, 48 % (n = 27) had received prior anthracycline treatment and 46 % (n = 26) had received prior taxane treatment, 36 % (n = 20) had received both anthracycline and taxane, and 41 % (n = 23) were anthracycline- and taxane-naïve (Table 1). For Study 206 patients, mean age was 57.0 years. Median relative delivered dose intensity for eribulin treatment ranged from 98.7 to 98.9 % across the anthracycline- and/or taxane-pretreated subgroups. Median treatment duration was 19.1 weeks in anthracycline-pretreated patients, 16.1 weeks in taxane-pretreated patients, 21.4 weeks in anthracycline- and taxane-pretreated patients, and 27.0 weeks anthracycline- and taxane-naïve patients.

**Table 1:** Study 206 (Single-Agent Eribulin in HER2− MBC): demographics and baseline characteristics

	With prior anthracycline (n = 27)	With prior taxane (n = 26)	With prior anthracycline and taxane (n = 20)	Without prior anthracycline or taxane (n = 23)
Female, n (%)	27 (100)	26 (100)	20 (100)	23 (100)
Age (years)				
Mean (SD)	55 (10)	55 (11)	55 (11)	61 (11)
Age group, n (%)				
<50 years	9 (33)	9 (35)	7 (35)	3 (13)
50–65 years	13 (48)	11 (42)	8 (40)	10 (43)
>65 years	5 (19)	6 (23)	5 (25)	10 (43)
Age at diagnosis (years)				
Mean (SD)	49 (10)	52 (11)	52 (10)	57 (11)
Estrogen receptor (ER) status, n (%)				
+	17 (63)	13 (50)	11 (55)	22 (96)
–	10 (37)	13 (50)	9 (45)	1 (4)
Progesterone receptor (PR) status, n (%)				
+	18 (67)	13 (50)	12 (60)	19 (83)
–	9 (33)	13 (50)	8 (40)	4 (17)
HER2 status, n (%)				
+	0	0	0	0
–	27 (100)	26 (100)	20 (100)	23 (100)

*SD* standard deviation

Fifty-two patients were enrolled in Study 208 (eribulin plus trastuzumab in HER2+ MBC), of which, 21 % (n = 11) had received prior anthracycline treatment, 44 % (n = 23) prior taxane treatment, 17 % (n = 9) had received both anthracycline and taxane, and 52 % (n = 27) were anthracycline- and taxane-naïve (Table 2). Mean age was 58.7 years and median delivered relative dose intensity for eribulin treatment across all anthracycline- and taxane-pretreatment subgroups ranged from 93.8 to 98.5 %. Median treatment duration was 28.0 weeks in anthracycline-pretreated patients, 28.1 weeks in taxane-pretreated patients, 21.4 weeks in anthracycline- and taxane-pretreated patients, and 37.3 weeks in anthracycline- and taxane-naïve patients.

**Table 2:** Study 208 (Eribulin + Trastuzumab in HER2+ MBC): demographics and baseline characteristics

	With prior anthracycline (n = 11)	With prior taxane (n = 23)	With prior anthracycline and taxane (n = 9)	Without prior anthracycline or taxane (n = 27)
Sex, n (%)				
Female	11 (100)	23 (100)	9 (100)	26 (96)
Male	0	0	0	1 (4)
Age (years)				
Mean (SD)	57 (12)	60 (11)	58 (12)	58 (11)
Age group, n (%)				
<50 years	4 (36)	5 (22)	3 (33)	5 (19)
50–65 years	3 (27)	8 (35)	2 (22)	15 (56)
≥65 years	4 (36)	10 (43)	4 (44)	7 (26)
Age at diagnosis (years)				
Mean (SD)	51 (14)	56 (12)	54 (14)	58 (11)
Estrogen receptor (ER) status				
+	7 (64)	14 (61)	6 (67)	20 (74)
–	3 (27)	9 (39)	3 (33)	7 (26)
Not done	1 (9)	0	0	0
Progesterone receptor (PR) status				
+	5 (45)	10 (43)	4 (44)	11 (41)
–	5 (45)	13 (57)	5 (56)	16 (59)
Not done	1 (9)	0	0	0
HER2 status, n (%)				
+	11 (100)	23 (100)	9 (100)	27 (100)
–	0	0	0	0

*SD* standard deviation

Data regarding eribulin administration for Studies 206 and 208 including duration, relative dose intensity, and average treatment day dose can found in Additional file 1: Table S1.

### Efficacy outcomes

**Fig. 1:**
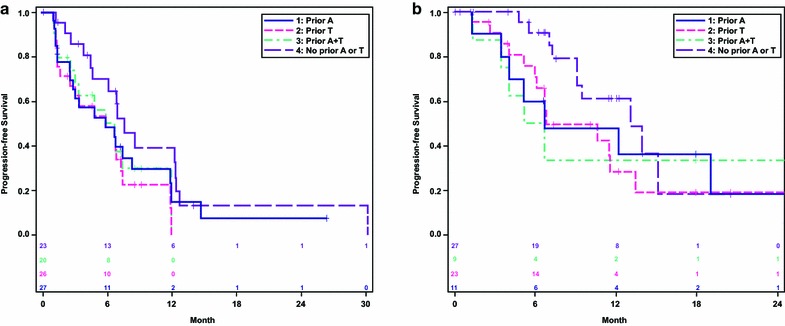
**a** Study 206 (Single-Agent Eribulin): Kaplan–Meier plot of progression-free survival by prior anthracycline (A) and/or taxane (T) treatment. **b** Study 208 (Eribulin + Trastuzumab): Kaplan–Meier plot of progression-free survival by prior anthracycline (A) and/or taxane (T) treatment

In Study 206, ORR and median PFS were 25.9 % and 5.8 months among patients who had received prior anthracycline, 26.9 % and 5.8 months among patients who had received prior taxane, 25.0 % and 6.7 months among patients who had received both anthracycline and taxane, and 30.4 % and 7.6 months among patients who were anthracycline- and taxane-naïve (Table 3; Fig. 1a).

**Table 3:** Best tumor responses for study 206 (HER2− MBC) and 208 (HER2+ MBC)

	Study 206 (HER2− MBC; Eribulin Only)				Study 208 (HER2+ MBC; Eribulin + Trastuzumab)			
	With prior anthracycline (n = 27)	With prior taxane (n = 26)	With prior anthracycline and taxane (n = 20)	Without prior anthracycline or taxane (n = 23)	With prior anthracycline (n = 11)	With prior taxane (n = 23)	With prior anthracycline and taxane (n = 9)	Without prior anthracycline or taxane (n = 27)
ORR, n (%)	7 (25.9)	7 (26.9)	5 (25.0)	7 (30.4)	7 (63.6)	13 (56.5)	5 (55.6)	22 (81.5)
95 % CI	(11.1, 46.3)	(11.6, 47.8)	(8.7, 49.1)	(13.2, 52.9)	(30.8, 89.1)	(34.5, 76.8)	(21.2, 86.3)	(61.9, 93.7)
CR, n (%)	0	0	0	0	2 (18.2)	2 (8.7)	1 (11.1)	0
PR, n (%)	7 (25.9)	7 (26.9)	5 (25.0)	7 (30.4)	5 (45.5)	11 (47.8)	4 (44.4)	22 (81.5)
PFS, median, m	5.8	5.8	6.7	7.6	6.7	6.8	5.9	13.1
95 % CI	(2.5, 8.3)	(1.6, 7.3)	(2.5, 11.9)	(4.6, 12.4)	(1.4, NE)	(6.0, 13.5)	(1.4, NE)	(9.1, NE)

*m* months, *NE* not estimable

In Study 208, ORR and median PFS were 63.6 % and 6.7 months among anthracycline-pretreated patients, 56.5 % and 6.8 months among taxane-pretreated patients, 55.6 % and 5.9 months among anthracycline- and taxane-pretreated patients, and 81.5 % and 13.1 months among anthracycline- and taxane-naïve patients (Table 3; Fig. 1b).

### Safety/tolerability

In Study 206, rates of serious TEAEs were similar in patients who had received prior anthracycline (29.6 %) and in those who had received prior taxane treatment (26.9 %); rates were 20.0 % in patients who had received both anthracycline and taxane, and 26.1 % in patients who were anthracycline- and taxane-naive. In Study 208, rates of serious TEAEs were 27.3 % in patients who had received prior anthracycline, 30.4 % in patients who had received prior taxane treatment, 33.3 % in patients who had received both anthracycline and taxane, and 29.6 % in patients who were anthracycline- and taxane-naive.

In Study 206, dose modifications due to AEs occurred at a similar rate in patients who had received prior anthracycline (66.7 %), prior taxane (65.4 %), or both anthracycline and taxane (70.0 %), but the rate was lower among patients who were anthracycline- and taxane-naïve (43.5 %). In Study 208, dose modifications occurred at a similar rate in patients who had received prior anthracycline (54.5 %), prior taxane (56.5 %), or both anthracycline and taxane (55.6 %), and were slightly higher in patients who were anthracycline- and taxane-naïve (63.0 %).

Rates of peripheral neuropathy and neutropenia are summarized in Table 4. In Study 206, rates of grade 2 (15.4–20.0 %) and grade 3 neuropathy (18.5–26.1 %) were generally similar, regardless of prior treatment (no grade ≥4 neuropathy was reported). The rate of neutropenia was lower among patients who were anthracycline- and taxane-naive (56.5 %), and was relatively similar among the other three groups (77.8–84.6 %). In Study 208, patients who had received taxanes had higher rates of grade 2 neuropathy (30.4 %) relative to the other groups (14.8–22.2 %), while those who were anthracycline- and taxane-naive had higher rates of grade 3 neuropathy (37.0 %) relative to the other groups (11.1–18.2 %); no grade ≥4 neuropathy was reported. The rate of neutropenia was higher among those who had received prior anthracyclines (81.8 %) and those who had received both prior anthracycline and taxane (77.8 %), somewhat lower among those who had received prior taxane (65.2 %), and lowest among those who were anthracycline- and taxane- naïve (51.9 %).

**Table 4:** Peripheral neuropathy and neutropenia AEs by prior anthracycline and/or taxane treatment

	Study 206 (HER2− MBC; Eribulin Only) (N = 56)				Study 208 (HER2+ MBC; Eribulin + Trastuzumab) (N = 52)			
	With prior anthracycline (n = 27)	With prior taxane (n = 26)	With prior anthracycline and taxane (n = 20)	Without prior anthracycline or taxane (n = 23)	With prior anthracycline (n = 11)	With prior taxane (n = 23)	With prior anthracycline and taxane (n = 9)	Without prior anthracycline or taxane (n = 27)
Peripheral neuropathy (SMQ), n (%)								
Grade 1	4 (14.8)	4 (15.4)	2 (10.0)	7 (30.4)	4 (36.4)	6 (26.1)	4 (44.4)	5 (18.5)
Grade 2	5 (18.5)	4 (15.4)	4 (20.0)	4 (17.4)	2 (18.2)	7 (30.4)	2 (22.2)	4 (14.8)
Grade 3	5 (18.5)	6 (23.1)	5 (25.0)	6 (26.1)	2 (18.2)	3 (13.0)	1 (11.1)	10 (37.0)
Grade ≥4	0	0	0	0	0	0	0	0
Febrile neutropenia	2 (7.4)	2 (7.7)	1 (5.0)	1 (4.3)	0	3 (13.0)	0	1 (3.7)
Neutropenia	21 (77.7)	22 (84.6)	16 (80.0)	13 (56.5)	9 (81.8)	15 (65.2)	7 (77.7)	14 (51.9)

## Discussion

First-line therapy for recurrent BC or MBC with eribulin (Study 206) or eribulin plus trastuzumab (Study 208) demonstrated antitumor activity with acceptable tolerability irrespective of prior anthracycline and/or taxane treatment. For Study 206, ORR was similar regardless of prior anthracycline and/or taxane treatment (ORR range: 25.0–30.4 %), although median PFS was slightly longer among patients who were both anthracycline- and taxane-naïve (7.6 months) relative to those who had received prior treatment (range 5.8–6.7 months). In Study 208, ORR was higher (81.5 %) and median PFS (13.1 months) was longer in patients who had not received prior anthracycline or taxane treatment compared to those who had received prior anthracycline and/or taxane treatment (ORR range 55.6–63.6 %; PFS range 5.9–6.8 months), suggesting that patients who are both anthracycline- and taxane-naïve have prolonged benefit from first-line eribulin plus trastuzumab.

Results for Study 206 presented here are comparable to those obtained with single-agent anthracyclines or taxanes. In a phase 3 study that compared doxorubicin and pegylated liposomal doxorubicin (N = 509), ORRs were 38 and 33 %, respectively, with 25 % of patients in each group having stable disease (SD), and a PFS of 7.8 and 6.9 months, respectively (O’Brien et al. 2004). In an analysis of individual patient data from studies using first-line single-agent anthracycline (doxorubicin) or taxane therapy (paclitaxel or docetaxel), ORRs were 38 % with anthracyclines and 33 % for taxanes (*P* = 0.08), and SD of 37 and 40 %, with a median PFS of 7.2 and 5.1 months, respectively (Piccart-Gebhart et al. 2008).

In Study 208, eribulin plus trastuzumab as first-line treatment for patients with HER2+ locally advanced BC or MBC demonstrated anti-tumor activity comparable to those reported for other chemotherapy combinations evaluated in this setting. Studies of trastuzumab plus docetaxel demonstrated ORRs ranging from 45 to 61 % and PFS ranging from 8.3 to 12.4 months (Hurvitz et al. 2013; Servitja et al. 2012; Andersson et al. 2011; Marty et al. 2005). A study that compared vinorelbine plus trastuzumab vs docetaxel plus trastuzumab reported an ORR of 59.3 % in both arms and time to progression of 15.3 and 12.4 months, respectively (Andersson et al. 2011). A study of combined oral or IV vinorelbine with trastuzumab reported an ORR of 70.3 % and time to progression of 9.3 months (Heinemann et al. 2011). The combination of capecitabine plus trastuzumab has resulted in an ORR of 38–65 % and PFS of 7.8 months (Yamamoto et al. 2008; Michalaki et al. 2010). ORRs from 36 to 75 % and PFS ranging from 7.1 to 9.9 months were demonstrated in trials combining trastuzumab and paclitaxel (Fountzilas et al. 2001; Gasparini et al. 2007; Robert et al. 2006). For the combination of trastuzumab plus paclitaxel and carboplatin, a PFS of 10.7 months and an ORR of 52 % was reported (Robert et al. 2006). However, with the large improvement in median OS observed with the addition of pertuzumab to docetaxel and trastuzumab as first-line treatment for HER2+ MBC (Baselga et al. 2012; Swain et al. 2013, 2014), the combination of trastuzumab plus pertuzumab and docetaxel (or paclitaxel) is recommended as the preferred standard of care (National Comprehensive Cancer Network 2015). The NCCN guidelines include trastuzumab alone or with docetaxel, vinorelbine, capecitabine, or paclitaxel; with or without carboplatin, as other first-line options (National Comprehensive Cancer Network 2015).

In both studies, tolerability was generally similar for chemotherapy-naïve patients and patients who had received prior anthracyclines and/or taxanes. In Study 206, the rate of serious TEAEs among chemotherapy-naïve patients was similar to the rates among anthracycline-pretreated patients and taxane-pretreated patients, although the rate was lower among patients who had received both anthracycline and taxane treatment. In Study 208, rates of serious TEAEs were comparable for chemotherapy-naïve and patients with prior anthracycline and/or taxane treatment. In both studies, rates of neuropathy (any grade) were similar among the subgroups of patients who had received prior anthracycline and/or taxane treatment; the rate among chemotherapy-naïve patients was greater than the rate among pretreated patients in Study 206 and was comparable to the rate among pretreated patients in Study 208. In both studies, rates of grade 2 and 3 neuropathy (no grade ≥4 neuropathy was reported) among patients who had received prior taxane treatment were comparable to or less than the rates among chemotherapy-naïve patients.

## Conclusions

As first-line therapy, eribulin in patients with HER2−MBC and eribulin plus trastuzumab in patients with HER2+ MBC was effective with acceptable tolerability, regardless of prior anthracycline or taxane treatment. In Study 206 (patients with HER2− MBC receiving eribulin only), ORRs and median PFS duration were similar in patients who had received prior anthracycline treatment and/or prior taxane therapy, although patients who were both anthracycline- and taxane-naïve had slightly longer median PFS. For Study 208 (patients with HER2+ MBC receiving combination eribulin/trastuzumab), ORR was higher and median PFS was longer among anthracycline- and taxane-naïve patients treated with eribulin plus trastuzumab compared with patients who had received prior anthracycline and/or taxane treatment. These data demonstrated that eribulin is an active agent in chemotherapy-naïve as well as in anthracycline- and/or taxane-pretreated patients with MBC.

## Additional file

**Additional file 1: Table S1.** Eribulin Administration Throughout Study 206 and Study 208.

## Abbreviations

AE: adverse event
BC: breast cancer
CR: complete response
DOR: duration of response
FAS: full analysis set
HER2: human epidermal growth factor receptor 2
HER2−: human epidermal growth factor receptor 2-negative
HER2+: human epidermal growth factor receptor 2-positive
MBC: metastatic breast cancer
ORR: objective response rate
OS: overall survival
PFS: progression-free survival
PR: partial response
SD: stable disease
TEAE: treatment-emergent adverse event
