# Supplementary material for: Impact of prior anthracycline or taxane use on eribulin effectiveness as first-line treatment for metastatic breast cancer: results from two phase 2, multicenter, single-arm studies
Source: Springerplus. 2015 Sep 21;4:532. doi: 10.1186/s40064-015-1322-y (PMC4577494; doi:10.1186/s40064-015-1322-y)
Supplement: Supplementary file 1 — Additional file 1: Table S1. Eribulin Administration Throughout Study 206 and Study 208. [file 40064_2015_1322_MOESM1_ESM.doc]

Supplemental Table 1 Eribulin Administration Throughout Study 206 and Study 208

|  | **Study 206 (HER2− MBC; Eribulin Only)** | | | | **Study 208 (HER2+ MBC; Eribulin + Trastuzumab)** | | | |
| --- | --- | --- | --- | --- | --- | --- | --- | --- |
| **Prior Anthracycline/**  **Taxane Use** | **With Prior Anthracycline**  **(n=27)** | **With Prior**  **Taxane**  **(n=26)** | **With Prior Anthracycline and Taxane**  **(n=20)** | **Without Prior Anthracycline or Taxane**  **(n=23)** | **With Prior Anthracycline (n=11)** | **With Prior**  **Taxane**  **(n=23)** | **With Prior Anthracycline and Taxane**  **(n=9)** | **Without Prior Anthracycline or Taxane**  **(n=27)** |
| **Duration** |  |  |  |  |  |  |  |  |
| Median, weeks | 19.1 | 16.1 | 21.4 | 27.0 | 28.0 | 28.1 | 21.4 | 37.3 |
| Min, Max | 3.1, 119.1 | 1.1, 50.1 | 3.1, 50.1 | 1.1, 130.1 | 0.1, 114.3 | 0.1, 114.3 | 0.1, 114.3 | 4.0, 94.1 |
| **Relative Dose Intensity: Core Treatment** |  |  |  |  |  |  |  |  |
| Median | 98.9 | 98.7 | 98.8 | 99.1 | 98.5 | 93.8 | 97.7 | 98.4 |
| Min, Max | 64.6, 101.3 | 68.6, 101.3 | 68.6, 101.3 | 47.6, 100.6 | 50.4, 101.4 | 50.4, 101.4 | 50.4, 101.4 | 71.7, 102.5 |
| **Relative Dose Intensity** |  |  |  |  |  |  |  |  |
| Median | 94.8 | 94.7 | 96.5 | 95.9 | 97.7 | 90.9 | 96.7 | 92.4 |
| Min, Max | 60.1, 101.3 | 60.1, 101.3 | 60.1, 101.3 | 47.9, 102.5 | 50.4, 101.4 | 50.4, 101.4 | 50.4, 101.4 | 54.2, 102.5 |
| **Average Daily Dose Intensity/Patient** |  |  |  |  |  |  |  |  |
| Median | 0.1 | 0.1 | 0.1 | 0.1 | 0.1 | 0.1 | 0.1 | 0.1 |
| Min, Max | 0.1, 0.2 | 0.1, 0.4 | 0.1, 0.2 | 0.1, 0.4 | 0.1, 1.4 | 0.1, 1.4 | 0.1, 1.4 | 0.1, 0.2 |
